# The effects of lockdown during the COVID-19 pandemic on fetal movement profiles

**DOI:** 10.1186/s12884-024-06259-8

**Published:** 2024-01-11

**Authors:** N. Reissland, B Ustun, J. Einbeck

**Affiliations:** 1 Department of Psychology, Durham University, Science Site, South Road, Durham, UK; 2 Division of Human Nutrition and Health, Wageningen University & Research, Stippeneng 4, Wageningen, 6708 WE The Netherlands; 3 Department of Mathematical Sciences, Durham University, Durham, UK

**Keywords:** COVID-19 pandemic lockdown, Pregnancy, Mental health, Fetal movement profile, Natural light exposure

## Abstract

**Purpose:**

The current study investigated the direct impact of the COVID-19 lockdown on fetal movements, addressing a critical research gap. While previous research has predominantly examined the effects of lockdown on maternal health and postnatal outcomes, little attention has been paid to the direct consequences on fetal well-being as indicated by their movement profile.

**Methods:**

We conducted analysis of movement profiles in 20 healthy fetuses during the COVID-19 pandemic lockdown (third national UK lockdown period between January and March 2021) and compared them with 20 healthy fetuses from pre-covid pregnancies, all at 32 weeks gestation. We controlled for maternal stress, depression, and anxiety.

**Results:**

Pregnant mothers during pre-covid compared with those during the COVID-19 lockdown reported similar levels of stress (*p* = 0.47), depression (*p* = 0.15), and anxiety (*p* = 0.07). Their fetuses, however, differed in their movement profiles with mouth movement frequencies significantly higher during COVID-19 lockdown (COVID-19 lockdown: mean of 5.909) compared to pre-Covid pregnancies (mean of 3.308; *p* = 0.029). Furthermore, controlling for maternal anxiety a regression analysis indicated that frequency of fetal mouth movements (*p* = 0.017), upper face movements (*p* = 0.008), and touch movements (*p* = 0.031) were all significantly higher in fetuses observed during lockdown compared to fetuses before the Covid period.

**Conclusion:**

Fetuses show an effect of lockdown independent of maternal anxiety, stress, or depression. These findings contribute to our understanding of fetal development during extraordinary circumstances, raising questions about the potential effects of having to stay indoors during lockdowns.

## Background

 Research on the impact of the COVID-19 pandemic on fetal movement profiles during the UK lockdown periods is notably scarce. While existing studies have primarily focused on the direct effects of COVID-19 infections on pregnant women and subsequent postnatal outcomes in their offspring [1], the potential consequences of lockdown measures on fetal well-being have received less attention. Although research regarding maternal infections with SARS-CoV-2 during pregnancy found an association with preterm birth and fetal death [1]; others investigating SARS-Covid effects on fetal neuro-sonographic abnormalities or cortical brain development found no significant evidence of such impact [2].

Postnatal research on the effects of maternal Covid infections investigated fidgety movements, which are small, erratic movements of the limbs, in 3–5 months old infants of mothers who had been exposed to COVID-19 [3]. These movements are crucial for assessing neuromotor development in early infancy [3]. They reported that the absence of fidgety movements in those infants indicated a higher risk for neurological disorders compared to a control group [3]. Examining the potential differential impact of the virus on male or female infants, Bordt et al. [4] examined the variations in immune responses to the SARS-Covid virus based on fetal sex and found that male fetuses showed less immunity transferred by the mother compared to female fetuses. Furthermore, Veeranna [5] highlighted effects of COVID-19 on the fetal auditory system, with increased neural delay of processing auditory stimuli, thereby demonstrating the wide-ranging effects of maternal Covid infections on fetal and neonatal development.

In contrast to these studies, our investigation focuses on whether pregnancy during lockdown periods has an impact on fetal movement profiles. These profiles encompass a range and frequency of movements a fetus typically exhibits, including mouth-, lower face-, upper face-, head turn-, touch- movement and eye blinks frequencies, and which are key indicators of healthy development [6, 7]. Deviations from the expected profile of fetal movements can signal fetal stress and developmental issues [8–12]. We hypothesized that the lockdown period may affect pregnant women, both psychologically and environmentally, thereby impacting fetal health [6]. Specifically, during the COVID-19 lockdown where government rules stated that one should not leave the house for more than one hour per day this period had the potential for impacting healthy fetal development during the COVID-19 lockdown [13].

While studies have investigated the effects of COVID-19 infections on pregnancy outcomes, as exemplified by the comprehensive review by Wastnedge et al. [14], the direct evidence of fetal effects during lockdowns remains largely unexplored. The need to study the impact of COVID-19 lockdowns on pregnancy is underscored by the fact that, while viruses can detrimentally affect fetal development [14], normal pregnancies involve an adapted maternal immune response that could be affected by the lockdown [15]. Additionally, the stress associated with a COVID-19 pregnancy, including the risk of preterm delivery, stillbirth, and increased delivery-related mortality, have a significant implication for maternal and fetal health [16]. This interpretation is supported by a large Swedish cohort study [17], which found an increased risk of autism and depression in offspring following viral infections during pregnancy, as well as studies correlating maternal stress and anxiety with neuropsychiatric diagnoses in offspring [18, 19].

In summary, our research addresses a significant gap by comparing fetal movement profiles during the COVID-19 lockdown with matched samples assessed before the pandemic, controlling for maternal stress, depression, and anxiety. This approach allows us to evaluate whether the lockdowns had additional effects on fetal development beyond the established impact of maternal mental health.

## Methods

### Ethical approval

This study was performed in accordance with the Declaration of Helsinki and ethical permission for the research reported in this paper was granted by Durham University (PSYCH-2020-12-07T13:46:58-dps0nr). All mothers gave informed written consent to participate and for publication.

### Study design

This study investigated potential effects of the lockdown period during COVID-19 on fetal movement profiles in the last trimester of pregnancy. To achieve our objectives, we employed a cohort study design, comprising two groups: an experimental group of pregnant women who underwent ultrasound scans during the COVID-19 lockdown period, and a control group of pregnant women from the same area whose ultrasound scans were conducted before the pandemic.

### Participants

We recruited a total of 40 pregnant participants whose fetuses (21 females, 19 males) were healthy at the 20-week anomaly scan (see Tables 1 and 2). All participants resided in the same geographical region in the north of England, were non-smokers, aged between 18 and 37, and had a healthy BMI ranging from 18 to 33. Exclusion criteria included any medication usage, recreational drug use, or diagnosed medical or mental health conditions. The experimental group of mothers during the COVID-19 lockdown period did not show any signs or symptoms of Covid for the past 14 days prior to their scans.

**Table 1:** Descriptive statistics of participants who were pregnant during the COVID-19 lockdown

	N	Minimum	Maximum	Mean	Std. Deviation
Maternal age	20	21.00	36.00	30.00	4.35
Gestational age	20	31.3	33.0	32.02	0.54
Maternal BMI	20	18.00	33.00	25.44	4.38
HADS anxiety	20	2.00	11.00	5.65	3.19
HADS depression	20	1.00	6.00	3.55	1.79
PSS perceived stress	20	0.00	23.00	11.50	6.20
MAAS Attachment	20	69.00	90.00	79.50	6.21

**Table 2:** Descriptive Statistics of participants who were pregnant prior to the COVID-19 pandemic

	N	Minimum	Maximum	Mean	Std. Deviation
Maternal age	20	18.00	37.00	27.55	5.62
Gestational age	20	31.0	33.00	31.98	0.60
Maternal BMI	5	19.50	29.00	24.98	3.45
HADS anxiety	20	0.00	9.00	3.90	2.61
HADS depression	20	0.00	8.00	2.65	2.05
PSS perceived stress	20	0.00	20.00	10.10	5.78
MAAS Attachment	20	70.00	90.00	81.80	5.97

### Data collection

Data for the experimental group were collected during the third UK national lockdown period, which spanned from January to March 2021. This period was characterized by a range of COVID-19 control measures and restrictions including stay-at-home orders, closures of non-essential businesses, school closures, social distancing requirements, face covering mandates, remote work, travel restrictions, limitations on social gatherings, and the initiation of a vaccination rollout.

Pregnant women underwent 4D ultrasound scanning at 32 weeks gestation for about 20 min following the guidelines of the British Medical Ultrasound Society [20] in a COVID-19 compliant clinic. Mothers completed the Hospital Anxiety and Depression Scale - HADS [21], Perceived Stress Scale – PSS [22], and Maternal Antenatal Attachment Scale - MAAS [23]. The HADS asks mothers to describe their feelings over the past week. It has two subscales, HADS-A (Anxiety/ 7 questions) and HADS-D (Depression/ 7 questions), and a total score range of 0 (minimum) to 21 (maximum). Anxiety statements are such “I get sudden feelings of panic”. Depression statements are such “I still enjoy the things I used to”. The PSS asks mothers to evaluate stress levels during the month prior to each ultrasound scan. It has 10 items, with answers based on 5-point Likert (ranging from 0 = ‘never’ to 4 = ‘very often’ stressed). In addition to the psychological assessments, we collected information about COVID-19 status (had Covid, no Covid), information not available for control group participants who underwent ultrasound scans before the pandemic.

### Coding of ultrasound scans

All ultrasound scans were coded frame by frame using the Observer X15T software programme (Wageningen, Netherlands). Coding included identifying various behavioural groups following the Fetal Observational Movement System (FOMS) guidelines [24]. Behavioural groups included mouth movements, lower face movements, upper face movements, head turn, touch movements and eye blinks. Each behavioural group was coded separately, with distinct start and stop codes corresponding to the visibility of the behavioural group. For example, the total duration of the mouth being visible throughout the scan is calculated via start codes when the mouth is visible and stop codes when the mouth cannot be coded. We repeated this process for each behavioural group. The relative frequency of each behaviour was computed due to variations in the duration of visibility among fetal scans.

### Reliability

To ensure the reliability of coding, 10% of the ultrasound scans were randomly selected and coded by a second, independent coder who was blinded to the study’s hypotheses and participant condition. Inter-rater reliability was assessed using Cohen’s kappa statistics, with a mean kappa value of 0.87 (range 0.83 to 0.95), indicating substantial agreement between coders.

### Statistical analysis

A rigorous statistical approach was employed to analyse the data [25], which will be fully described in the Results section. This included appropriate statistical tests or models to test research hypotheses. Potential confounding variables were accounted for through statistical controls.

## Results

### Maternal mental health comparison

**Fig. 1:**
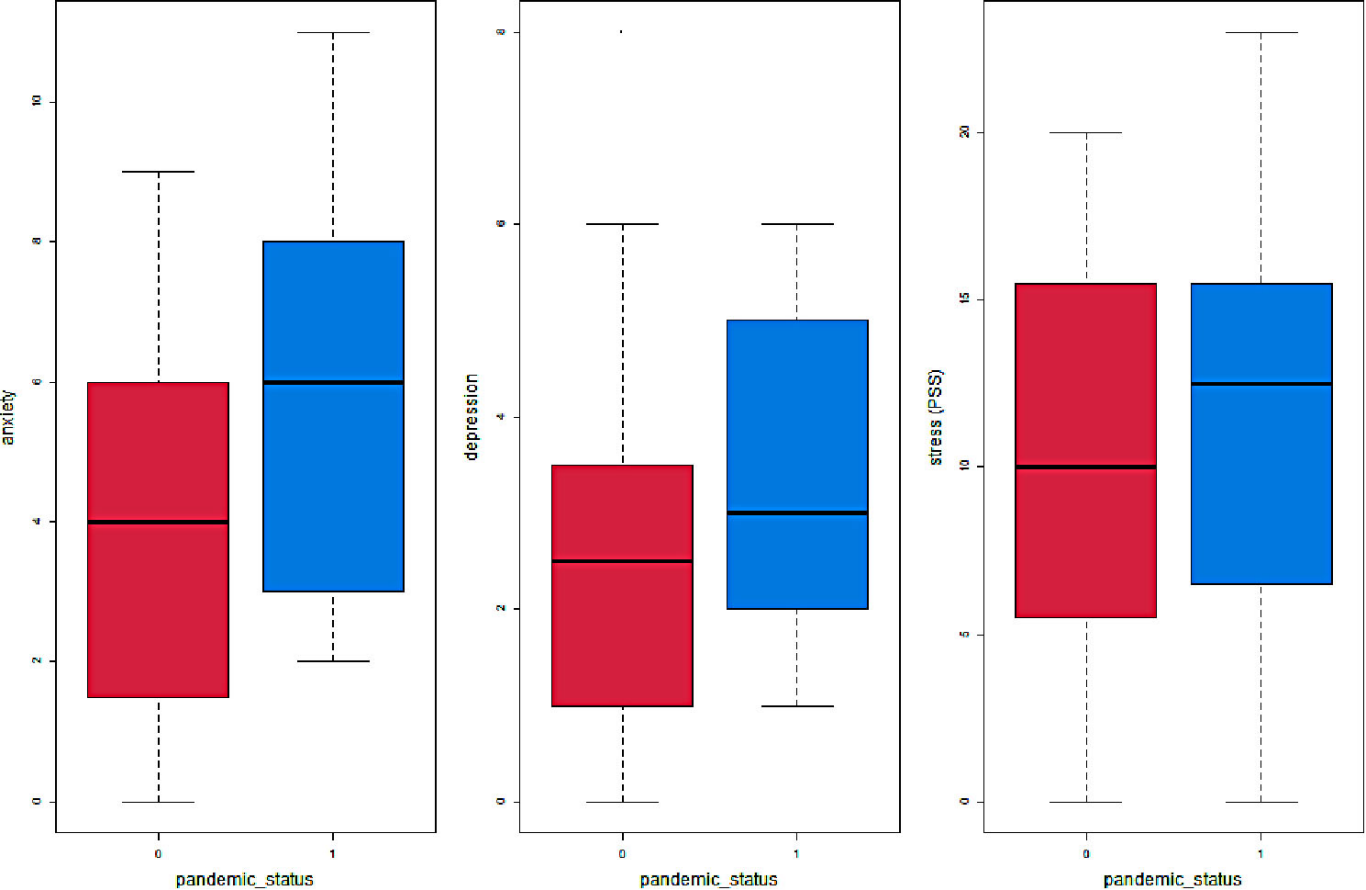
Boxplots of mental health indicators by lockdown status (0 = control; 1 = experimental)

Using a two-sided, two-sample t-test (assuming unequal variances and with the Welch correction of degrees of freedom), we found no significant differences between the experimental (mean = 11.5) and control groups (mean = 10.1) in relation to Perceived Stress (*p* = 0.47), depression (experimental mean = 3.55; control mean = 2.65, *p* = 0.15), and anxiety (experimental mean = 5.65; control mean = 3.9. *p* = 0.07). The relationship between the pandemic lockdown status and the maternal mental health indicators is illustrated in Fig. 1.

### Comparison of movement frequencies

**Fig. 2:**
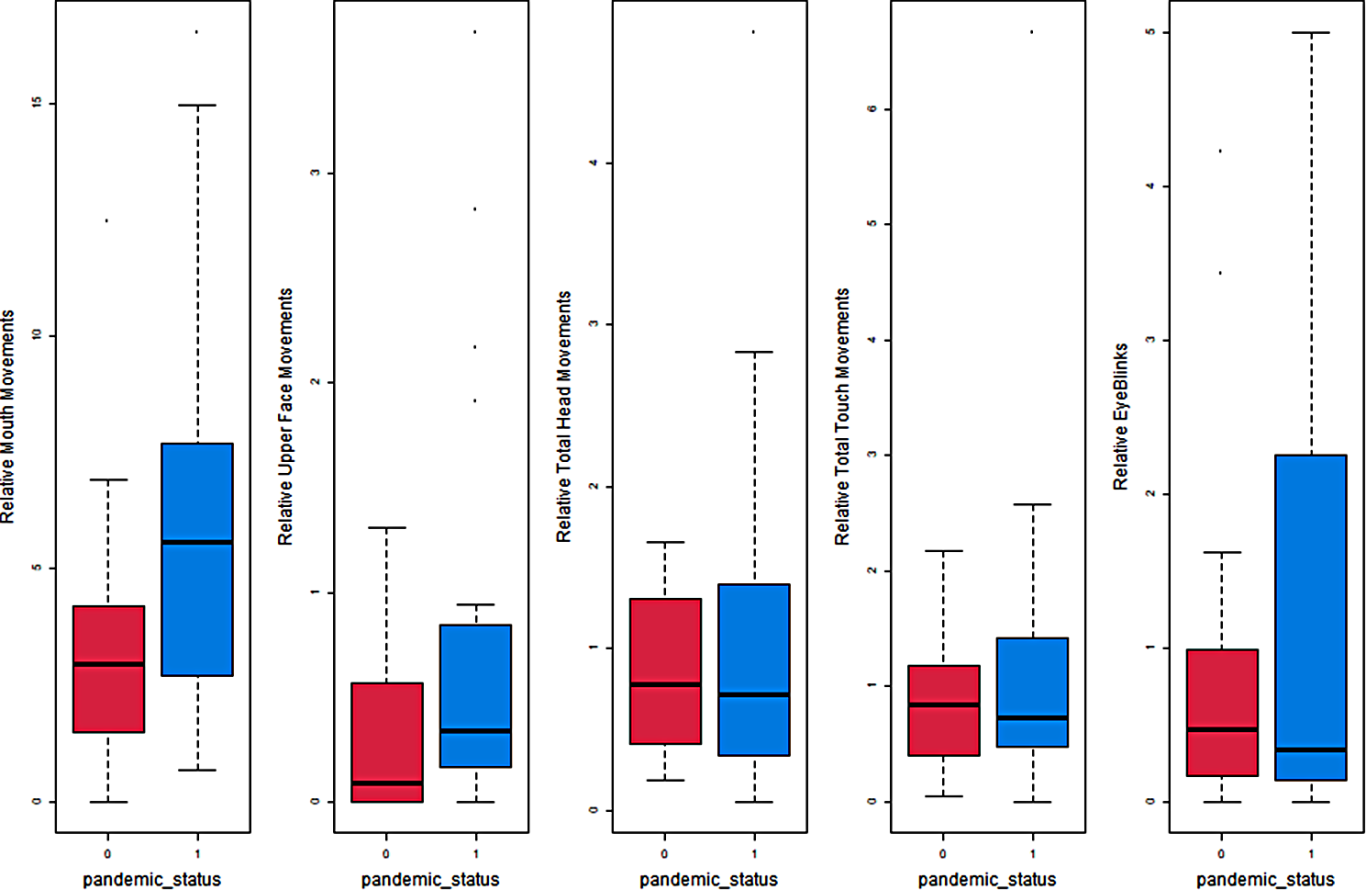
Boxplots of relative number of movements for all behavioural groups by lockdown status (0 = control; 1 = experimental)

For each of the behavioural groups (mouth movements, lower face movements, upper face movements, head turn, touch movements, and eye blinks), we carried out two-sample t-tests using the Welch corrected degrees of freedom. Each sample consists of *n* = 20 values representing the “number of movements divided by codable scan length”, referred to as relative number of movements. These relative movement numbers are summarized in Fig. 2. Results of the comparisons of relative number of movements by treatment status are summarized in Table 3. We found a significant difference in mean values between the control and experimental groups for mouth movements, with experimental fetuses exhibiting significantly more movements. A similar trend of increased relative number of movements from the pre-Covid to the Covid sample, was consistent across all behavioural groups, statistical significance was not reached at the 5% level except for mouth movements.

**Table 3:** Means of the number of movements relative to codable scan length

	Mean (controls)	Mean (experimental)	t-value (abs)	*p*-value
Mouth movements	3.308	5.909	2.291	**0.029**
Upper face movements	0.304	0.776	1.882	0.071
Head turns	0.841	1.058	0.794	0.435
Touch movements	0.895	1.212	0.888	0.383
Eye blinks	0.833	1.200	0.843	0.405

Note: Significant results at the 5% level highlighted in bold

### Model-based analysis

To further investigate the effects of the pandemic on movement profiles, we conducted a regression analysis for each of the five movement types, adjusting for covariates. The models included binary predictor variables for lockdown status (0 = control, 1 = experimental), fetal gender (0 = female, 1 = male), continuous predictor variables (anxiety, depression, perceived stress, maternal attachment), gestational age (weeks), and maternal age (years). Negative binomial regression models with log-link were used to account for overdispersion, with a codable scan length for the respective movement types entering as an offset. That is, the models take shape of type log(E(movement count)) = log(codable scan length) + ${\beta }_{0}+ {\sum }_{j}{\beta }_{j}{x}_{j}$ where “movement count” is the count (an integer value) of the respective movement type, E(.) stands for the expectation operator, log(codable scan length) is the offset, ${\beta }_{0}$ is the intercept parameter, and ${\beta }_{j}$ are the coefficients for predictor variable ${x}_{j}$, which correspond to the variables listed in the first column of Table 4 beneath the intercept. Due to the presence of overdispersion, a Negative Binomial Type 2 model was used for the modelling of all behavioural groups.

Results of the fitted models are summarized in Table 4. While the overall trends align with the two-sample t-tests, adjusting for covariates revealed a significant impact of the lockdown on both upper face and mouth movements. Additionally, increasing gestational age was associated with fewer head turns tends (*p* = 0.016). We omit the results for the fitted models of eye blinks due to the absence of significant effects (see Fig. 2). It is important to note that, from a strict statistical perspective, one could argue for the consideration of multiple testing, given that we are examining five outcome variables concurrently. However, even after applying a Bonferroni correction, which sets the significance threshold at 1% instead of 5%, the significant effect of the lockdown on upper face movements remains statistically significant.

### Mediation analysis for the effect of anxiety on mouth movements

A mediation analysis was conducted to explore whether anxiety mediated the effect of the lockdown. For example, whether it could be the case that the lockdown has caused anxiety in mothers and this anxiety then leads to a change of frequency in movements. This analysis focused on Upper Face movements, Mouth movements, and Touch movements, as these had shown significant impact in previous analyses. We specifically examined anxiety as a possible mediator, given its close-to-borderline significant effect observed.

The mediation analysis aims to determine whether inclusion of the mediator (anxiety) in the model altered the significance of the lockdown’s effect. If this inclusion results in a loss of significance of the effect of the pandemic, this would indicate a mediation effect. Removal of anxiety from the models did not notably alter the significance of the lockdown coefficient for mouth movements (*p* = 0.016) but increased the *p*-value for Upper Face movements (*p* = 0.013) and Touch movements (*p* = 0.033). Thus, *inclusion* of anxiety would not decrease significance of the effect of the lockdown. This suggests that anxiety did not mediate the impact of the lockdown on the movement frequency.

**Table 4:** Coefficient estimates and *p*-values *(in brackets and italic)* for the behavioural groups

Coefficient	Mouth movements	Upper Face movements	Total touches	Head turns
Intercept	6.123 *(0.465)*	8.799 *(0.525)*	1.031 *(0.915)*	14.712 ***(0.044)***
Pandemic status	0.621 ***(0.017)***	1.203 ***(0.008)***	0.667 ***(0.031)***	0.267 (*0.267)*
Fetus gender	-0.054 *(0.823)*	-0.015 *(0.972)*	-0.294 *(0.333)*	-0.200 *(0.393)*
Anxiety	-0.016 *(0.841)*	-0.288 ***(0.046)***	0.131 *(0.200)*	-0.016 *(0.831)*
Depression	0.065 *(0.444)*	0.059 *(0.677)*	-0.062 *(0.540)*	0.027 *(0.731)*
Stress	-0.002 *(0.958)*	0.025 *(0.656)*	-0.054 *(0.229)*	-0.033 *(0.248)*
Attachment	0.003 *(0.892)*	-0.080 ***(0.038)***	0.036 *(0.198)*	-0.025 *(0.263)*
Gestational age	-0.043 *(0.218)*	-0.029 *(0.602)*	-0.032 *(0.410)*	-0.073 ***(0.016)***
Maternal age	0.008 *(0.741)*	-0.015 *(0.724)*	-0.006 (0.856)	-0.008 *(0.724)*

Note: Significant results (at the 5% level) are highlighted in bold face. Results for Eye blinks are omitted due to the lack of significant effects

## Discussion

The current study found similar levels of anxiety, stress, and depression reported by mothers tested pre-COVID-19 and during the COVID-19 lockdown. However, fetal movement profiles of the two groups differed significantly. This result suggests that other factors apart from maternal mental health may influence fetal movement profile differences observed. Therefore, it is essential to explore changed movement profiles and the effects on fetal health in more detail. Although, research suggest that maternal mental health plays a role in the effects of the COVID-19 lockdown on the pregnancy, we did not find this in our sample when testing general anxiety in the two samples. However other potential explanations include less exposure to daylight, given the mandate of staying at home which affects mental health as well as sleep [26; 27]. One study [28] cited that most time was spent in the home (74%), and only 8% of time was spent outdoors.

Although we did not test, in our study, for the effects of hormonal factors other research indicates significant alterations in thyroid hormone levels among pregnant women during the COVID-19 pandemic [29]. Given that during lockdown mothers were required to stay indoors, they were potentially exposed to low levels of natural daylight and increased artificial light sources. The disruption of circadian rhythms and hormonal functions caused by altered lighting conditions has the potential to impact fetal development significantly [30]. Hence, we might speculate that during the COVID-19 lockdown, changes in daily routines and time spend indoors may have influenced melatonin production, impacting thyroid regulation and thereby changing fetal movement patterns.

Despite the limitations imposed by the relatively small sample size, our study offers an important insight into the effects of lockdown conditions on fetal movements. The challenges (restrictions on 4D ultrasound scans) imposed by lockdown conditions made it difficult to access a larger pool of participants, resulting in a limited dataset. The COVID-19 pandemic unfolded with varying degrees of severity and lockdown measures across different regions and time periods. Our study focused on a specific period during the third UK national lockdown between January and March 2021. While we have highlighted the potential influence of time spent indoors and hormonal factors, our study did not assess these variables. Future research should aim to quantify environmental factors, such as natural light exposure and artificial light sources, to better understand their role in fetal development.

## Conclusion and implications

In conclusion, our study highlights the complex relationship of influences shaping fetal movements during the lockdown period of the COVID-19 pandemic. It highlights the need for a holistic approach to understand the multifaceted effects of quarantine conditions on the delicate process of fetal development, including time spend indoors, maternal mental health, and potentially hormonal factors. Future research needs to follow up infants who have been born in this period, assessing their socio-emotional and adaptive behaviours, as well as their cognitive and language development. These insights can inform evidence-based clinical practices and public health strategies to better manage the challenges and uncertainties that pregnant women and their unborn children face in the context of a global pandemic.

## Data Availability

The data are available at: https://www.maths.dur.ac.uk/users/jochen.einbeck/Data/Fetal-Lab/.
